# Assessing the Acute Effects of Accentuated Eccentric Contrast Training on Vertical Jump Using Wireless Dual Force Plates in Young Basketball Players

**DOI:** 10.3390/s26041159

**Published:** 2026-02-11

**Authors:** Jorge Clemente-Benedicto, Carlos García-Sánchez, Jaime González-García, Diego Alonso-Aubin, Raúl Nieto-Acevedo

**Affiliations:** 1 Deporte y Entrenamiento Research Group, Departamento de Deportes, Facultad de Ciencias de la Actividad Física y del Deporte (INEF), Universidad Politécnica de Madrid, Calle Martín Fierro 7, 28040 Madrid, Spain; jorgeclementebene@gmail.com; 2 Exercise and Sport Sciences, Faculty of Health Science, Universidad Francisco de Vitoria, 28223 Pozuelo, Spain; jaime.gonzalez@ufv.es; 3 Performance Area, Royal Spanish Football Federation, 28232 Las Rozas, Spain; 4 Faculty of Heath Sciences HM Hospitals, University Camilo José Cela, C/Castillo de Alarcón, 49, Villanueva de la Cañada, 28692 Madrid, Spain; diegoalexandre.alonso@ucjc.edu; 5 HM Hospitals Health Research Institute, 28015 Madrid, Spain; 6 Strength Training and Neuromuscular Performance Research Group (STreNgthP), Faculty of Health Sciences HM Hospitals, University Camilo José Cela, C/Castillo de Alarcón, 49, Villanueva de la Cañada, 28692 Madrid, Spain; 7 Facultad de Ciencias Biomédicas y de la Salud, Universidad Alfonso X el Sabio (UAX), Avenida de la Universidad, 1, Villanueva de la Cañada, 28691 Madrid, Spain; racevnie@uax.es

**Keywords:** strength training, accentuated eccentric loading, postactivation performance enhancement, plyometric training, athletic performance

## Abstract

Background: Basketball performance depends strongly on physical preparation. A novel approach is accentuated eccentric loading within contrast training, though its acute effects using dumbbells remain underexplored. Methods: Twelve youth basketball players (age = 16.0 ± 0.3 years; body mass = 81.5 ± 7.6 kg) completed three sessions with dumbbell loads equivalent to 15%, 30% and 45% BW. CMJ performance was measured using dual wireless dual force plates. Assessments were conducted before the protocol and at 3, 9, and 15 min post intervention. Subjective responses were collected via wellness, RPE and readiness questionnaires. A two-way repeated measures ANOVA with Bonferroni corrections was applied, and the significance level was set to α = 0.05. Results: Significant decreases in jump height (*p* = 0.010) and average propulsive power (*p* = 0.005) were observed in the 45% BW condition at 3 and 9 min. Jump momentum decreased significantly at 30% and 45% BW at 3 and 9 min (*p* = 0.010; *p* = 0.033). No significant differences were detected in other CMJ force–time metrics (*p* > 0.05). Conclusions: Dumbbell-loaded CMJs as an accentuated eccentric loading contrast exercise did not produce generalized improvements but induced acute decreases at higher loads. However, they may still be useful in individual cases for athletes with favorable responses after monitoring.

## 1. Introduction

Within a popular and widely practiced sport like basketball [1], increasing importance is being given to physical fitness [2], as it allows players to improve their performance [3] and protects against potential sports injuries derived from competition and the high load of matches throughout the season [4]. The main adaptations derived from a properly designed traditional strength training program include increases in muscle cross-sectional area, greater motor unit recruitment, improvements in inter and intramuscular coordination, enhanced muscle–tendon stiffness and reductions in muscle inhibition [2]. This may enhance athletes’ capacities, including maximal dynamic and isometric strength, reactive capacity and the ability to apply force per unit of time, a phenomenon known as Rate of Force Development (RFD) [2].

In terms of strength and conditioning training, there is a method called complex training [5]. This training methodology has four variations and each of them combines at least two biomechanically similar exercises in the same session using different loads [5]. One of them is contrast training, which consist of alternating heavy concentric loads (85% 1 RM) and light concentric loads (body mass to 60% 1 RM) in the same set [5]. The main characteristic of this training method is that it proposes very differentiated training stimuli, the first generating the highest force productions at low contractile velocities and the second producing force at high velocities [6]. This differentiation in the type of activations provided to the velocity of the exercise is essential to generate adaptations in the trained contractile velocity [7] thus generating positive adaptations in maximal strength, RFD, force production at high velocities, muscle power and hypertrophy [8].

Coaches can use other training methodologies and implement them with their athletes, such as eccentric training. In this type of stimulus, the contraction occurs as a result of applying less, but still controlled, force than the one the muscle is facing [9], which can produce up to 50% more force than in the concentric phase [10]. The main benefits of this training include increased cross-sectional area, fiber elongation, and greater pennation angle. These adaptations stimulate type II fibers and enhance performance factors such as speed, acceleration, and maximal strength [11]. Accentuated eccentric loading is a type of eccentric training variation that allows the eccentric phase of movement to be overloaded, while the concentric phase uses a lower load that the subject can lift [10]. It is important that, when repetitions are done, there are no interruptions, employing clamps, rubber bands or chains that allow overloading the exercise without wasting time [10]. The main benefit of this training system is that the stretching–shortening cycle is present, which allows combining the phases and producing greater eccentric and concentric forces [12], activating the IIx fibers to a greater extent, enhancing strength and power, and improving performance in the vertical jump and throws [13]. Regarding the effects of the accentuated eccentric loading methodology, improvements have been found in the following performance factors: vertical jump and one-legged jump [14], change in direction [15], maximal lower body strength [16] and horizontal jump [17]. This training methodology can produce acute improvements in athletic performance due to an increase in the storage of elastic energy during the eccentric phase and greater activation of motor neurons [18].

This improvement in strength, power or speed after a conditioning contraction that is usually seen in jumping or running exercises is known as Post-Activation Potentiation Enhancement (PAPE) [19]. This effect is defined as an enhancement of the contractile response and allows the total recruitment of muscle fibers [20]. The PAPE effect is not only achieved through conditioning contractions or maximum voluntary contractions, it can also be obtained as a result of increasing factors such as: muscle temperature, metabolism, basal oxygen consumption, muscle activation or motor learning. The PAPE event occurs over a period of 6 to 10 min [5]. The effect caused depends on each subject and factors such as their strength level or distribution of muscle fibers [21]. PAPE can even influence other aspects of the athlete, changing the psychological state of the subjects [18]. It should be added that immediately after making a conditioning contraction, the athlete has a drop in performance where the athlete is not able to apply the same force as before [22]. This is produced by the fatigue induced by the exercise, but the effect of PAPE duration is greater than the window where this fatigue is present [22]. It is essential to take this aspect into account to benefit from the effects of this phenomenon [20].

To the best of our knowledge, the most similar research exposed 40 male physical education students to an 8-week training program. The aim was to investigate the chronic effects of using dumbbells as an accentuated eccentric loading in the countermovement jump (CMJ) [18]. Positive enhancements were found in the 1 RM squat and jump height, but no significant differences were found in *T*-tests or 30 the m sprint.

Therefore, the aim was to analyze the acute effects of using dumbbells in the CMJ as an accentuated eccentric loading in contrast training on vertical jump performance. Based on previous research, we hypothesize that using lighter loads produces higher peak power [18], while heavier loads will exert the greatest eccentric forces on the CMJ [23].

## 2. Materials and Methods

### 2.1. Participants

G*Power software (version 3.1.9.7, Kiel University, Schleswig-Holstein, Kiel, Germany) was used to calculate an estimation of the sample size for matched pairs. It showed that a sample size of 15 would be enough when considering a moderate effect size of 0.5, an α of 0.05 and a statistical power of 0.8, assuming a moderate correlation and nonsphericity correction of 1 [24]. However, the number of players on the team prevented reaching the sample size calculated by G*Power. Twelve basketball players participated voluntarily in this study (age = 16.0 ± 0.3 years; height = 192.5 ± 7.6 cm; body mass = 81.5 ± 7.6 kg), with 9.0 ± 1.8 years of basketball experience and 3.8 ± 1.0 years of strength training experience. A convenience sampling approach was used due to participant accessibility and suitability. As inclusion criteria to be able to participate, two necessary premises were set: having experience in strength training for at least 2 years, and not having any injury that might prevent completion of the training protocol. In addition, as they were minors, both the players and a legal guardian signed an informed consent form allowing the use of their data for the purpose of the study. The study was approved by the Ethics Committee of Camilo José Cela University, Spain (protocol code 16_23_RNM_FP) and conducted in accordance with the Declaration of Helsinki.

### 2.2. Design

A randomized crossover and counterbalanced design was used to investigate the acute effects of the use of dumbbells of different percentages of body weight (BW) in contrast training on the CMJ. This is a quasi-experimental study, since it does not have a control group, and group assignment was not randomized.

#### Training Protocol

**Figure 1:**
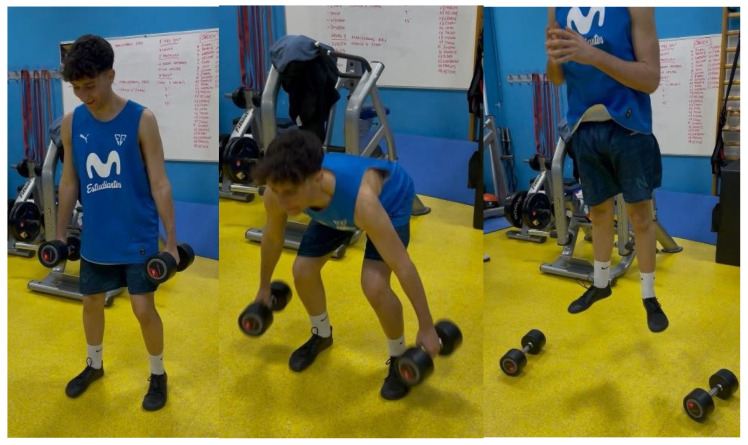
Complete sequence of performing an accentuated eccentric loading jump during the training protocol.

**Figure 2:**
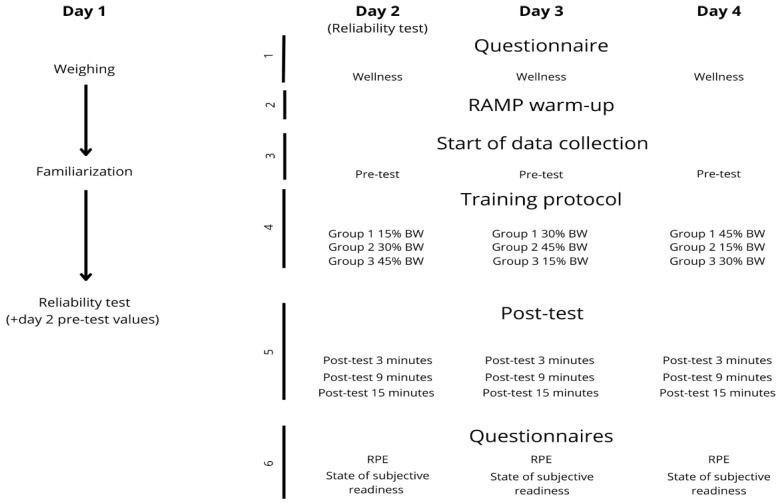
Overview of the experimental design and testing protocol. Pre-test and post-test consisted of CMJ; training protocol consisted of eccentric loading training of CMJ with dumbbells.

Data collection was carried out over 4 weeks, always on the same day, ensuring a one-week break between measurements. The first session was used for familiarization with the testing procedures and protocol methodology and to assess, with the pre-test data from day 2, test–retest reliability, where the players performed a total of 3 jumps after the warm-up and before starting the training protocol with loads. The intervention consisted of 3 sets of 6 repetitions with the dumbbells, releasing them at the lowest position of movement, and 4 repetitions without weight in any phase of movement, with a short rest of 10 s between exercises and a recovery of 3 min between sets. Figure 1 includes explanatory photographs of how the repetitions were performed. Participants were grouped based on their body weight. One group used two dumbbells representing 15% BW, another 30% BW and the last 45% BW [23]. The post-test data were taken at 3, 9 and 15 min after the intervention ended. Variables such as diet or external factors of the subject that may influence the study have not been taken into account. The entire protocol is graphically summarized in Figure 2.

### 2.3. Testing Procedures

All test sessions were preceded by a brief explanatory introduction, where the protocol, the number of jumps they had to perform at each moment in the data collection and the loads that each group had to use were explained. Then, subjects performed a standardized warm-up, which followed a Raise, Activate, Mobilize and Potentiate (RAMP) structure, starting with lateral movements and light jogging to raise body temperature, followed by activation exercises such as front lunges, then exercises such as dynamic quadriceps stretches for mobility and, finally, for potentiation, maximum jumps with a total duration of 15 min [25].

Countermovement test (Figure 2: numbers 3 and 5): Wireless dual force plates were placed on the flat, level ground and calibrated before each trial (zeroed) [26]. Subjects remained completely upright and motionless for at least one second, during which time their weight and mass were measured. Once completed, a light and sound signal indicated the start of the jump [27]. These instructions were given to the athletes: “start fully upright and with the hands on the hips, start a downward movement, and immediately after jump to get the highest height as fast as possible” [28,29]. The repetitions the subjects performed during the test directly depended on the timing, making three attempts in the pre-test, and only two in the different post-test measurements, to ensure times were consistent and accurate to the correct minute. Between repetitions, subjects had at least 10 s of rest. If the jump was performed incorrectly, the attempt was discarded and repeated immediately afterwards.

Wellness questionnaire (Figure 2: number 1): Participants completed the wellness questionnaire each morning via a Google Form. It was used to get the athlete’s individual and subjective perception of: fatigue, sleep quality, muscle pain, stress level, and recovery status. A Likert scale was used in this questionnaire, where all variables were rated with a number from 1 to 5. A rating of 1 indicates “very low” and 5 represents “very high” [30]. Recovery status metrics followed the same methodology as the others variables, but used a scale from 1 to 10 [31].

Rate of Perceived Exertion (RPE) (Figure 2: number 6)*:* This form provides information about the degree of subjective effort that the protocol has required. A 10-point Borg scale was used, where 1 indicates “very light activity” and 10 represents “maximal effort” [30]. It was completed right after the protocol using a Google Form [32].

Perceptual questionnaire (Figure 2: number 6): A Google Form focusing on the subjective state of preparedness was done after the protocol. The aim of it is to analyze the effects that the training protocol had on the following variables: physical condition, physical performance, aggressiveness, fatigue level, muscle heaviness, and enhancing effect [33]. All subjects responded with a number from 1 to 10, where 1 indicates “no impact” and 10 represents “great impact” [33].

### 2.4. Data Analyses

Wireless dual force plates (Hawkin Dynamics Inc., Westbrook, ME, USA), sampling at 1000 Hz [34] were used to measure countermovement jumps. Data analysis was performed automatically after the test using Hawkin Dynamics, Inc.’s proprietary software, in which forward dynamics were applied to calculate various performance force-time metrics related to acceleration, velocity, and displacement [26].

Manufacturer’s guidelines have been used to determine CMJ phases, divided into: the un-weighting phase, braking phase, propulsive phase, flight phase and landing phase [27]. All force–time metrics were calculated automatically by Hawkin Dynamics, Inc.’s proprietary software and represent the person (analysis of participant’s body mass), outcome (referring to jump performance), driver (underpinning relative forces applied during the propulsive and braking phases) and strategy (how the jump was executed) [35]. Force–time metrics selected are included and briefly explained in Table 1 [34]. For the analysis, an average of the values obtained in each measurement was made [36].

### 2.5. Statistical Analysis

All data are presented as the mean ± SD. For statistical analysis, Jeffreys’s Amazing Statistics Program (JASP) open-source software was used (version 0.95.3, University of Amsterdam, Amsterdam, The Netherlands). The normality assumption was tested using the Shapiro-Wilk test. Relative between-session reliability was calculated using the intraclass correlation coefficient (ICC, 3,1k) plus 95 CIs and interpreted based on the lower bound 95 CI (−95 CI) as ≥0.90 = excellent, 0.750–0.899 = good, 0.500–0.749 = moderate, and <0.50 = poor [37]. The coefficient of variation (CV) was used to provide absolute reliability, where values below 10% were considered data within an acceptable and reliable threshold [26]. Standard Errors of Measurement (SEMs) and their associated 95% CIs, as well as the smallest detectable change (SDC), were calculated to establish error values between testing sessions [38].

The significance level was set to α = 0.05. A two-way repeated measures ANOVA was conducted considering two factors, the time of data collection (3, 9 and 15 min), and the condition (15%, 30% and 45% BW). Partial eta squared ($\eta_{p}^{2}$) was calculated for group effects with the following interpretation: >0.010–0.059 = small, >0.060–0.139 = moderate, and >0.140 = large [39]. The effect size (ES) of Cohen’s d was calculated and interpreted following the above threshold guidelines: <0.20 = trivial, 0.20–0.59 = small, 0.60–1.19 = moderate, 1.20–1.99 = large, 2.00–4.00 = very large and >4.00 = extremely large [26].

Post hoc comparisons were corrected for multiple testing using the Bonferroni method to find significant differences. When correcting for multiple testing, the JASP software adjusts the *p*-values in the outputs to normal behavior, being directly compared with alpha. Individual response analysis was calculated using the Smallest Worthwhile Change (SWC) (SWC = 0.2 × between-subject SD) [40].

## 3. Results

The reliability data from the CMJ test are presented in Table 2. All CMJ force–time metrics displayed excellent reliability (ICC = 0.91–1.00) based on the 95% CI lower bound with acceptable variability (CV = 0.68–4.54).

Pre–post results are presented in Table 3, Table 4 and Table 5. The two-way repeated measures ANOVA (Moment*Condition) did not show a significant interaction effect in jump height (*p* = 0.335, $\eta_{p}^{2}$ = 0.105), jump momentum (*p* = 0.165, $\eta_{p}^{2}\;$= 0.137), mRSI (*p* = 0.269, $\eta_{p}^{2}$ = 0.115), average propulsive power (*p* = 0.851, $\eta_{p}^{2}$ = 0.042), peak propulsive power (*p* = 0.532, $\eta_{p}^{2}$ = 0.079), propulsive impulse (*p* = 0.479, $\eta_{p}^{2}$ = 0.046), braking impulse (*p* = 0.802, $\eta_{p}^{2}$ = 0.048) or time to takeoff (*p* = 0.085, $\eta_{p}^{2}$ = 0.164).

**Figure 3:**
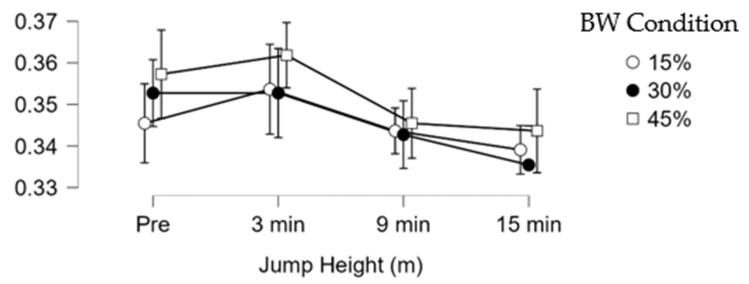
Jump height (m) change during testing.

**Figure 4:**
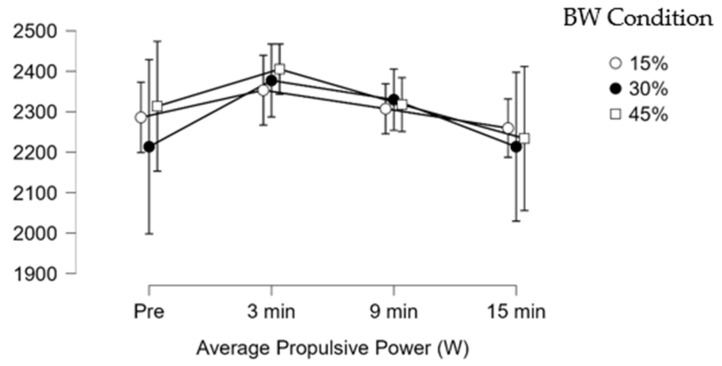
Average propulsive power (W) change during testing.

**Figure 5:**
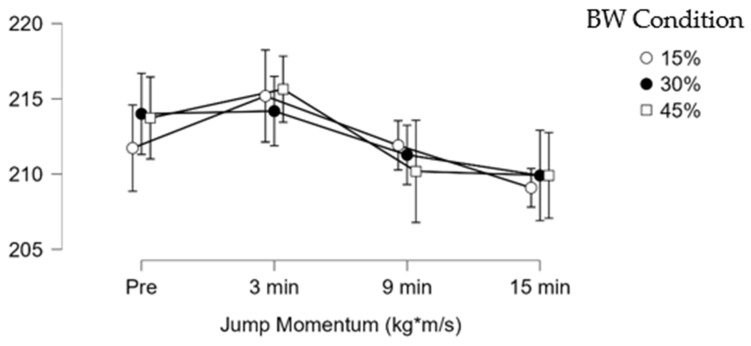
Jump momentum (kg*m/s) change during testing.

Post hoc analysis revealed significant decreases and small effect size in jump height in the 45% BW condition between 3 min (0.36 ± 0.05 m) and 9 min (0.35 ± 0.05 m) (mean difference (Δ) = −0.01 m; *p* = 0.010; ES = 0.33). Significant decreases and small effect sizes were observed in average propulsive power in the 45% BW condition between 3 min (2405.00 ± 200.80 W) and 9 min (2318.00 ± 203.80 W), (Δ = −87.00 W; *p* = 0.005; ES = 0.29). Significant decreases in the jump momentum force-time metric with trivial effect sizes were observed in the 30% BW condition between 3 min (214.20 ± 23.83 kg*m/s) and 9 min (211.30 ± 23.96 kg*m/s), (Δ = −2.90 kg*m/s; *p* = 0.010; ES = 0.12). Significant decreases with small effect sizes in the 45% BW jump momentum force–time metric were found at 3 min (215.60 ± 21.28 kg*m/s) and 9 min (210.20 ± 20.48 kg*m/s) (Δ = −5.40 kg*m/s; *p* = 0.033 (Bonferroni-adjusted); ES = 0.23). The other CMJ force–time metrics did not show significant differences between conditions or times (*p* > 0.05). Figure 3, Figure 4 and Figure 5 illustrate the change along testing of three different force-time metrics.

Analysis of positive individual response between baseline and post-test and SWD values are presented in Table 6. The values obtained in the wellness form show results of 3.09 ± 0.72 a.u. for fatigue, 3.42 ± 0.78 a.u. for sleep quality, 2.99 ± 0.77 a.u. for muscle soreness, 3.69 ± 0.88 a.u. for stress level and 6.41 ± 1.74 a.u. for recovery status. The RPE form indicates that the degree of effort in the session was 5.91 ± 1.13 sRPE with 15% BW, 5.91 ± 0.83 sRPE with 30% BW and 5.91 ± 1.92 sRPE with 45% BW. No significant differences were found in the perceptual questionnaire in any of the variables measured based on the load used (*p* > 0.05).

## 4. Discussion

The aim of this research was to analyze the acute effects of using dumbbells as an accentuated eccentric loading in contrast training on neuromuscular performance. To the of our knowledge, this is the first study to use this methodology and this selection of exercises. The main findings that: (a) it is a training method that does not produce kinematic or kinetic changes regardless of the moment or condition, although it can be beneficial in specific cases; (b) in individual cases, it has positive effects on subjective readiness; and (c) the RPE does not change depending on the load. Regarding our hypotheses, none of them were confirmed, as lighter loads did not produced significant changes in peak power, nor did higher loads have produced greater eccentric forces.

Previously published research reported similar results to our investigation, as no significant differences were found when using accentuated eccentric loading in back squats on CMJ at 3, 6, 9 and 12 min (*p* > 0.05) [41]. The sample, as in our research, was made up of basketball players, but the sample size was larger, as they had 15 subjects (age = 15.60 ± 1.10 years; body mass = 78.30 ± 19.60 kg; height = 182 ± 7.13 cm). Strength levels could explain these results, as some studies indicate that athletes with lower strength levels may benefit from using higher eccentric loads (120–130% 1 RM), while on the other hand, stronger individuals may obtain greater benefits from using lighter eccentric loads (110% 1 RM) [42,43] due to specific neuromuscular adaptations [44]. This is very useful information that allows coaches to individualize exercise prescriptions to increase athletic performance. In addition, and in line with our results, acute significant decreases were found when accentuated eccentric loading and two different rest redistribution conditions were used in back squat exercises on the CMJ jump (*p* < 0.05) [45]. One reason for these results could be the number repetitions, since rest redistribution methodology allows us to accumulate a greater volume per set and accentuated eccentric loading generates larger muscle damage [10]. So controlling, adjusting and monitoring training variables such as load, intensity, volume and methodology will be fundamental to achieve the desired effect. For this, it is essential to take into account all the variables mentioned, adjusting them to the athlete’s context and monitoring whether significant improvements occur.

Similarly, some studies have used different tools than in our investigation. In this case, a contrast training protocol was implemented for 8 weeks, where the accentuated eccentric loading was achieved using a flywheel machine, and the ballistic exercise consisted of a drop jump [46]. There is no correspondence in the results with our research, reporting a significant increase in CMJ height (*p* = 0.008) and maximal strength (*p* = 0.001) force–time metrics. The possible explanation for these results could be due to neural factors, such as an enhanced stretch-shortening cycle, increased excitability of the Golgi tendon organs or changes in muscle coordination [46]. These results could be a consequence of myogenic factors, including an increase in squat strength, which directly influence CMJ performance [46]. Coaches should consider these aspects when designing training programs. On the other hand, there are studies that investigate the acute effects of the implementation of a flywheel machine [16]. In contrast to our research, significant acute effects were found on the CMJ height at 3 min (*p* = 0.002), 7 min (*p* = 0.022) and 9 min (*p* = 0.002). Peak power force–time metrics also improved, and significant differences were found at 3 min (*p* = 0.009), 5 min (*p* = 0.02), 7 min (*p* = 0.011) and 9 min (*p* = 0.008). The main reason that may explain these results could be the selection of exercises, since the flywheel machine generates greater resistance during the entire range of movement [47]. Similarly, it should be emphasized that each exercise produces a different effect, and enabling the possibility of creating a more favorable space to obtain better benefits and maximize performance both acutely and chronically [42]. Consequently, the selection of exercises by the coach is essential to achieving the desired effects on the athletes’ performance.

Positive enhancements, contrary to our findings, have been found when accentuated eccentric loading is used in the CMJ for 8 weeks. In this case dumbbells representing loads between 10 and 40% BW were used [18]. Significant differences were found in the 1 RM back squat (*p* < 0.01) and CMJ (*p* < 0.01). These results may be due to proper technique, as the exercise must be performed correctly, without interruptions between the eccentric and concentric phases. Therefore, the long duration of the protocol can be very beneficial for athletes, allowing them to learn and consolidate the movement and thus maximize the benefits of this method [18]. Another possible explanation for the results obtained could be the strength level of the sample [42], because athletes with higher strength levels benefit more from this protocol than those with lower levels. Furthermore, the basketballers samples are usually used to ballistic and plyometric training, but not so much to resistance training.

Finally, it was challenging to compare the results of this study with previous research due to methodological differences. None of the mentioned articles includes a wellness questionnaire or an individual responses analysis. In fact, only two include a reliability test, which makes it impossible to determine whether the changes observed in those studies represent real improvements in the athletes’ performances [18,41]. The first investigation that utilized a reliability test, used Kistler wireless dual force plates and reported excellent reliability (ICC = 0.94–0.99) but unacceptable variability (CV = 11.80–20.40%). The comparison of values suggests that the measurements were not performed correctly, since data are unreliable because the CV values are greater than the 10% threshold previously mentioned. Improper use of the measurement tools makes it difficult to compare data, an aspect that must be taken into account when interpreting the values. In the second one, the results are not in line with ours, showing good-to-excellent reliability (ICC = 0.80–0.90) and not acceptable variability (CV = 13.60–21.10%) in jump heigh, jump momentum and peak propulsive force force-time metrics. As in our study, Hawkin Dynamics wireless dual force plates were used in this investigation. This underscores the importance of rigor when using these types of technologies to obtain valid and reliable data. On the other hand, only one study included RPE form [46], showing no significant differences between the protocols of their study (*p* > 0.05), and showing higher values than in our research (7.10 ± 1.60 sRPE).

Although the current study provides useful information for coaches and strength and conditioning professionals, some limitations should be mentioned: first, the sample size, including only 12 players, is not enough to achieve the calculated statistical power. Secondly, the number of familiarization sessions is another limitation, as it may have had a direct influence on the correct execution of the exercises. Third, we were not informed about the athletes’ nutrition during the protocol or the influence of external factors such as other commitments that could have directly influenced the results obtained.

The present results could be used by strength and conditioning coaches to optimize the physical performance of players before competitions, as it is another tool available since it does not harm performance and can have positive effects in individual cases. The individualization of training programs therefore gains importance, making it possible to get the most benefit from each athlete by adapting the training protocol to the individual context and circumstances. Future research reducing per-set volume, extending rest time, including a larger sample size and stronger athletes or incorporating female basketball players would elucidate the acute effects of using dumbbells as an accentuated eccentric loading in contrast training. In addition, future research should examine the effects of monitoring external variables such as diet, rest, stress or exercises familiarization time.

## 5. Conclusions

In this preliminary study with a small sample, using dumbbells as an accentuated eccentric loading within contrast training did not induce statistically significant group-level improvements in kinematic or kinetic performance force–time metrics. The number of subjects, since it is smaller than that suggested by G*Power, may reflect an inability to detect real effects in the training protocol. This protocol may serve as an individualized training tool to optimize neuromuscular readiness without inducing fatigue. Monitorization and communication between the athlete and physical trainer will be essential to optimize the effects that this training method can produce.

## Figures and Tables

**Table 1:** Explanatory and classification of the force–time metrics selected for analysis.

Category	Metric	Explanatory Brief
Person	Body Mass (kg)	Subject’s body weight
Outcome	Jump Height (m)	Vertical height achieved
	Jump Momentum (kg*m/s)	The vertical momentum of the system center of mass at the instant of take-off
	mRSI (m/s)	Ratio obtained by dividing the jump height by the time until takeoff.
	Avg. Propulsive Power (W)	The average mechanical power applied to the system center of mass during the propulsion phase.
	Peak Propulsive Power (W)	The peak instantaneous mechanical power applied to the system
Driver	Propulsive Impulse (N.s)	The vertical impulse applied to the system center of mass during the propulsion phase
	Braking Impulse (N.s)	The vertical impulse applied to the system center of mass during the braking phase
Strategy	Time To Takeoff (ms)	Total time taken from the initiation of movement to the take-off

Hawkin Dynamics incorrectly stated that mRSI units were “a.u.” instead of “m/s”.

**Table 2:** Reliability test of the CMJ force-time metrics selected. Test performed on days 1 and 2.

	Session 1 (Familiarization)			Session 2 (Day 2 Pre-Test)						
Category	Mean	±	SD	Mean	±	SD	ICC (95% IC)	CV (%)	SEM (95% CI)	SDC
Body mass (kg)	81.60	±	8.58	82.11	±	8.57	1.00 (0.99–1.00)	0.68	0.64	1.10
Jump height (m)	0.36	±	0.06	0.34	±	0.05	0.96 (0.88–0.99)	3.51	0.02	0.02
Jump momentum (kg*m/s)	215.45	±	25.20	211.64	±	23.09	0.99 (0.96–1.00)	1.66	4.06	7.19
mRSI (m/s)	0.51	±	0.08	0.48	±	0.06	0.91 (0.77–0.97)	4.54	0.03	0.04
Avg. Propulsive Power (W)	2392.82	±	276.15	2359.73	±	234.41	0.97 (0.90–0.99)	1.80	55.99	85.10
Peak Propulsive Power (W)	4235.73	±	560.70	4159.55	±	478.31	0.98 (0.96–0.99)	1.34	75.96	116.35
Propulsive Impulse (N.s)	432.55	±	56.85	424.27	±	58.25	0.96 (0.88–0.99)	2.79	13.94	23.27
Braking Impulse (N.s)	239.82	±	41.75	234.00	±	40.90	0.98 (0.95–0.99)	2.69	7.36	12.08
Time To Takeoff (ms)	725.98	±	57.63	716.82	±	55.45	0.91 (0.76–0.97)	2.35	18.95	34.32

SD: standard deviation; ICC: intraclass correlation coefficient; CV: coefficient of variation; SEM: standard error of the mean; SDC: smallest detectable change.

**Table 3:** Countermovement jump force–time metrics analysis (15% BW).

Condition	CMJ Force–Time Metrics	Pre			3 min			9 min			15 min			Cohen’s d pre vs. 3 min	Cohen’s d pre vs. 9 min	Cohen’s d 3 min vs. 9 min	Cohen’s d pre vs. 15 min	Cohen’s d 3 min vs. 15 min	Cohen’s d 9 min vs. 15 min
15% BW	Jump height (m)	0.345	±	0.05	0.354	±	0.05	0.344	±	0.05	0.339	±	0.05	−0.16	0.04	0.199	0.126	0.29	0.09
	Jump momentum (kg*m/s)	211.73	±	24.71	215.20	±	26.51	211.90	±	24.34	209.10	±	24.10	−0.15	−0.01	0.14	0.11	0.26	0.12
	mRSI (m/s)	0.47	±	0.07	0.49	±	0.07	0.48	±	0.07	0.47	±	0.08	−0.23	−0.13	0.10	0.01	0.22	0.12
	Avg. Propulsive Power (W)	2286.00	±	224.57	2353.00	±	271.50	2307.00	±	263.70	2259.00	±	267.70	−0.02	−0.07	0.15	0.09	0.31	0.16
	Peak Propulsive Power (W)	4155.55	±	494.21	4216.00	±	510.50	4123.00	±	501.10	4037.00	±	464.30	−0.12	0.06	0.18	0.23	0.34	0.17
	Propulsive Impulse (N.s)	432.82	±	62.18	433.80	±	56.09	431.30	±	58.75	433.00	±	62.24	−0.02	0.03	0.04	0.00	0.01	−0.03
	Braking Impulse (N.s)	244.36	±	53.95	246.90	±	44.56	243.90	±	50.31	247.60	±	51.53	−0.05	0.01	0.06	−0.07	−0.02	−0.08
	Time To Takeoff (ms)	743.18	±	56.93	717.80	±	67.09	721.50	±	57.70	725.50	±	78.83	0.37	0.31	−0.05	0.26	−0.11	−0.06

Significance level is indicated by the number of symbols: one symbol for *p* < 0.05, two for *p* < 0.01.

**Table 4:** Countermovement jump force–time metrics analysis (30% BW).

Condition	CMJ Force–Time Metrics	Pre			3 min			9 min			15 min			Cohen’s d pre vs. 3 min	Cohen’s d pre vs. 9 min	Cohen’s d 3 min vs. 9 min	Cohen’s d pre vs. 15 min	Cohen’s d 3 min vs. 15 min	Cohen’s d 9 min vs. 15 min
30% BW	Jump height (m)	0.353	±	0.04	0.353	±	0.05	0.343	±	0.05	0.335	±	0.05	0.00	0.20	0.20	0.34	0.34	0.14
	Jump momentum (kg*m/s)	214.00	±	24.41	214.20	±	23.83 ^^	211.30	±	23.96	209.90	±	25.65	−0.01	0.12	0.12	0.17	0.18	0.06
	mRSI (m/s)	0.49	±	0.05	0.49	±	0.07	0.49	±	0.09	0.48	±	0.08	0.00	−0.01	−0.01	0.13	0.13	0.15
	Avg. Propulsive Power (W)	2214.00	±	395.30	2377.00	±	263.70	2330.00	±	279.80	2214.00	±	436.10	−0.05	−0.39	0.16	0.00	0.54	0.39
	Peak Propulsive Power (W)	4210.00	±	458.00	4242.00	±	460.60	4201.00	±	497.70	3934.00	±	748.00	−0.06	0.02	0.08	0.53	0.59	0.51
	Propulsive Impulse (N.s)	433.20	±	59.69	437.80	±	62.40	426.00	±	50.12	428.50	±	68.46	−0.08	0.12	0.20	0.08	0.16	−0.04
	Braking Impulse (N.s)	244.90	±	43.37	246.60	±	49.37	242.20	±	41.92	246.00	±	53.12	−0.04	0.05	0.09	−0.02	0.01	−0.08
	Time To Takeoff (ms)	727.50	±	60.57	737.00	±	67.26	709.80	±	61.61	710.90	±	66.37	−0.14	0.26	0.39	0.25	0.38	−0.02

Significance level is indicated by the number of symbols: one symbol for *p* < 0.05, two for *p* < 0.01. ^ indicates significant differences vs. 9 min.

**Table 5:** Countermovement jump force–time metrics analysis (45% BW).

Condition	CMJ Force–Time Metrics	Pre			3 min			9 min			15 min			Cohen’s d pre vs. 3 min	Cohen’s d pre vs. 9 min	Cohen’s d 3 min vs. 9 min	Cohen’s d pre vs. 15 min	Cohen’s d 3 min vs. 15 min	Cohen’s d 9 min vs. 15 min
45% BW	Jump height (m)	0.357	±	0.04	0.362	±	0.05 ^^	0.345	±	0.05	0.344	±	0.05	−0.09	0.24	0.33	0.27	0.36	0.04
	Jump momentum (kg*m/s)	213.72	±	23.04	215.60	±	21.28 ^	210.20	±	20.48	209.90	±	22.30	−0.08	0.15	0.23	0.16	0.24	0.01
	mRSI (m/s)	0.50	±	0.06	0.51	±	0.08	0.49	±	0.09	0.49	±	0.08	−0.15	0.19	0.34	0.21	0.35	0.01
	Avg. Propulsive Power (W)	2314.00	±	271.30	2405.00	±	200.80 ^^	2318.00	±	203.80	2234.00	±	401.30	−0.31	−0.01	0.29	0.26	0.57	0.28
	Peak Propulsive Power (W)	42,390.00	±	449.20	4096.00	±	705.40	4120.00	±	420.90	4110.00	±	443.50	0.27	0.23	−0.05	0.25	−0.03	0.02
	Propulsive Impulse (N.s)	426.90	±	63.11	429.10	±	58.26	424.80	±	50.67	423.90	±	51.62	−0.04	0.04	0.07	0.05	0.09	0.02
	Braking Impulse (N.s)	236.20	±	41.92	239.50	±	41.59	238.00	±	42.20	248.00	±	55.68	−0.07	−0.04	0.03	−0.24	−0.18	−0.21
	Time To Takeoff (ms)	710.30	±	83.31	713.50	±	77.38	719.30	±	81.37	707.60	±	65.16	−0.05	−0.13	−0.08	0.04	0.08	0.17

Significance level is indicated by the number of symbols: one symbol for *p* < 0.05, two for *p* < 0.01. ^ indicates significant differences vs. 9 min.

**Table 6:** Analysis of positive individual response in 3 force–time metrics (comparison between baseline and moments).

				3 min			9 min		15 min		
Cond.	Force–Time Metrics	SWD	Pos.	Neu.	Neg.	Pos.	Neu.	Neg.	Pos.	Neu.	Neg.
15% BW	Jump height (m)	0.01	3	7	2	0	10	2	0	8	4
	Jump momentum (kg*m/s)	0.45	5	7	0	5	4	3	0	10	2
	mRSI (m/s)	0.01	2	10	0	0	12	0	1	10	1
30% BW	Jump height (m)	0.01	1	10	1	0	9	3	0	6	6
	Jump momentum (kg*m/s)	0.45	2	10	0	1	6	5	1	4	7
	mRSI (m/s)	0.01	1	10	1	1	10	1	1	10	1
45% BW	Jump height (m)	0.01	2	9	1	1	8	3	0	8	4
	Jump momentum (kg*m/s)	0.45	3	8	1	1	6	5	0	7	5
	mRSI (m/s)	0.01	1	11	0	1	10	1	1	10	1

## Data Availability

The original contributions presented in this study are included in the article. Further inquiries can be directed to the corresponding author.
